# The differential diagnosis of tiredness: a systematic review

**DOI:** 10.1186/s12875-016-0545-5

**Published:** 2016-10-20

**Authors:** Rebekka Stadje, Katharina Dornieden, Erika Baum, Annette Becker, Tobias Biroga, Stefan Bösner, Jörg Haasenritter, Christian Keunecke, Annika Viniol, Norbert Donner-Banzhoff

**Affiliations:** Department of General Practice/Family Medicine, University of Marburg, Karl-von-Frisch-Str. 4, 35043 Marburg, Germany

**Keywords:** Tiredness, Fatigue, Primary care, Meta-analysis

## Abstract

**Background:**

Tiredness is one of the most frequent complaints in primary care. Although often self-limiting and frequently associated with psychosocial stress, patients but also their physicians are often uncertain regarding a serious cause and appropriate diagnostic work-up. We conducted a systematic review and meta-analysis of studies reporting on differential diagnosis of fatigue in primary care.

**Methods:**

MEDLINE, EMBASE and conference abstracts were searched for primary care based studies of patients presenting with tiredness. Twenty-six studies were included. We report on anaemia, malignancy, serious organic disease, depression and the chronic fatigue syndrome (CFS) as causes of tiredness as presenting complaint.

**Results:**

We found considerable heterogeneity of estimates which was reduced by limiting our analysis to high quality studies. Prevalences were as follows-anaemia: 2.8 % (CI (confidence interval) 1.6–4.8 %); malignancy: 0.6 % (CI 0.3–1.3 %); serious somatic disease: 4.3 % (CI 2.7–6.7 %); depression 18.5 % (CI 16.2–21.0 %). Pooling was not appropriate for CFS.

In studies with control groups of patients without the symptom of tiredness, prevalence of somatic disease was identical to those complaining of tiredness. Depression, however, was more frequent among those with tiredness.

**Conclusions:**

Serious somatic disease is rare in patients complaining of tiredness. Since prevalence is similar in patients without tiredness, the association may not be causal. Extensive investigations are only warranted in case of specific findings from the history or clinical examination. Instead, attention should focus on depression and psychosocial problems.

**Electronic supplementary material:**

The online version of this article (doi:10.1186/s12875-016-0545-5) contains supplementary material, which is available to authorized users.

## Background

Tiredness is one of the most frequent complaints of patients in primary care. In a Dutch primary care registry, tiredness was the second most common presenting symptom [1]. In a Canadian study [2] 13.6 % of patients consulted for tiredness and for 6.7 % it was their main complaint. The figures are even higher when patients are systematically asked about the presence of tiredness [3–6].

Tiredness is usually self-limiting and easily explained by obvious circumstances, but sometimes it occurs in the context of defined somatic diseases, such as anaemia or hypothyroidism, or of mental disorder, such as depression or anxiety. The diagnosis chronic fatigue syndrome (CFS) implies a long duration and severe impairment of patients.

Primary care practitioners, who are usually the first health professionals to be contacted for tiredness, must decide on the kind and extent of diagnostic measures to take. This is fraught with uncertainty since serious disease may occur, but is rare and often untypical. Somatic investigations usually yield negative results and do not seem to clarify the underlying cause. For a rational work-up of these patients, knowledge of disease probabilities is an essential first step.

Although single studies of the frequency of somatic and mental illness in patients with tiredness have been conducted, no systematic review has been published so far.

We conducted a systematic review investigating the relevant aetiologies of tiredness in primary care.

## Methods

### Data sources and search strategy

We developed a search syntax with two components; one referred to the symptom of tiredness, the other to the setting of primary care. The detailed search syntax can be found in Additional file 1. We searched the electronic databases PubMed and EMBASE. Additionally, we carried out a manual search in the congress abstracts of the European General Practice Research Network and the North American Primary Care Research Group. Lastly, we checked references in articles already included.

### Inclusion and exclusion criteria

We included studies investigating tiredness in primary care settings. Studies from secondary or tertiary care were excluded. Exept from qualitative studies, we included all kind of study designs. Since our concern is tiredness as a clinical problem, we included only studies in which tiredness was presented as primary complaint or a second mentioned symptom. We thus excluded surveys in which patients were systematically interrogated about tiredness. We excluded studies in which only patients suffering from a certain illness, such as cancer, or patients with an increased probability for a certain illness were recruited. The studies had to provide estimates of the prevalence of at least one underlying disease. We included studies with study-specific data collection as well as those based on patients’ clinical records. Diagnoses associated with tiredness were retrieved from clinical records or reported by researchers. Patients could be identified prospectively by study physicians or retrospectively from clinical records.

### Study selection

Two authors (RS, KD) independently screened the titles and abstracts of identified references for relevance. Original publications with unselected patients from the primary care setting were reviewed as full texts independently by RS and KD. Disagreements regarding eligibility were resolved by discussion. If no consensus was reached a third author (NDB) was consulted.

### Data extraction

We extracted the following information for each eligible study: bibliographic data (author, publication year, title, journal), country, method of recruitment, definition of tiredness, study design characteristics, number of participating practices and/or physicians, sample size and response rate. The following were of particular relevance to underlying diseases: diagnostic categories/diagnoses, diagnostic work-up, prevalence rates of the diseases among the study population, definition of a control group without the symptom.

### Quality assessment

We assessed the quality of included studies according to the following criteria that were developed by our research group [7]: [1] Was the study prospective?; [2] were all patients with the symptom included (consecutive recruitment or random sample)?; [3] were inclusion and exclusion criteria precisely defined?; [4] was the study design multicentered?; [5] were the number of drop-outs and the reasons for withdrawal specified?; [6] were the diagnostic categories well-defined?; [7] was the diagnostic work-up to establish the diagnosis appropriate? [8] Was the diagnostic work-up applied to each patient? [9] Were the same diagnostic procedures applied to all patients?; [10] was there a control group without the symptom?. We scored these values with “yes”, “no”, “criteria fulfilled for part of the aetiologies” or “unclear”.

### Data synthesis

In the analysis we aimed to estimate how often the symptom tiredness is caused by a particular disease. For each study presenting data for a particular disease we calculated the respective proportion and 95 % confidence interval. We expected substantial between-study heterogeneity due to study design (methodological heterogeneity), definition of the symptom, of underlying conditions, diagnostic work-up, case-mix, health care system and time period (clinical heterogeneity). In order to visualize variation across studies, we grouped all eligible studies by underlying diseases and plotted proportions using forest plots. To quantify heterogeneity we calculated tau^2^ and 95 %-prediction intervals. In meta-analyses of interventions, tau^2^ estimates the between-study variance in a random-effects model [8]. Note that in our case the term ‘effect’ refers to a proportion of patients with a particular disease. The prediction interval can be interpreted as a range, in which the “true” proportion of a future study similar to those included in the analysis will lie with a probability of 95 %. It is therefore an intuitive measure to illustrate the heterogeneity across studies [8]. In order to identify causes of and account for heterogeneity we grouped studies according to predefined clinical and design criteria. We pooled only studies that had precisely defined diagnostic criteria and used an appropriate diagnostic work-up using a random effects model [9].

For the statistical computations and displays we used the statistical software R 2.14.0 (Foundation for Statistical Analysis, Vienna, Austria) and the R package ‘meta: Meta-Analysis with R’ [10].

## Results

### Search result and study selection

**Fig. 1:**
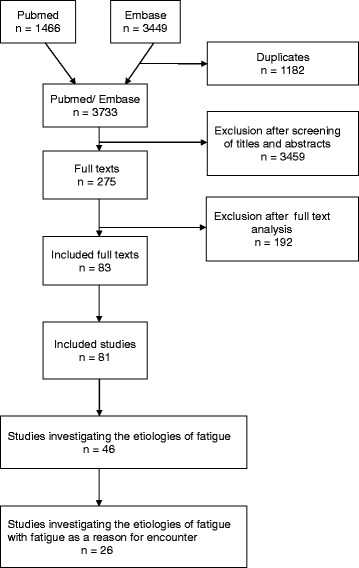
Flow of search

The electronic literature search was carried out in October 2010 and produced 3733 publications. 83 of them met our inclusion criteria. A few studies resulted in more than one publication so that we found 81 relevant studies overall. Forty-six studies dealt with the aetiology of tiredness. We excluded 20 studies in which patients were systematically asked about tiredness, the remaining 26 studies investigating tiredness as a reason for encounter or secondary complaint form the sample of our review. Further details of the selection process are given in a flow-chart (Fig. 1).

### Included studies

Most studies were conducted in Europe (*n =* 12). The number of included patients ranged from 22 to 10,279. Detailed information about the included studies is given in Table 1 and Additional file 2.

**Table 1:** Characteristics of the included studies

First author, year	Country	Sample size	Symptom definition	Anaemia	Malignancy	Severe somatic disease	Depression	CFS
Friedlander, 1962 [43]	USA	71	“Unbearable fatigue”					
Morrell, 1972 [29]	GB	58	New symptom that wasn’t brought up at a doctor’s visit during the last 12 months, main reason for encounter	X				
Morrison, 1980 [15]	USA	176	Cause unknown	X		X	X	
Jerrett, 1981 [16]	GB	300	Tiredness as a central problem (“tiredness”, “need of tonic”, “run down”, etc.)	X	X	X	X	
Sugarman, 1984 [17]	USA	118	Cause unknown	X		X	X	
Knottnerus, 1986 [44]	NL	174	New Symptom	X				
Nelson, 1987 [45]	USA	71	At least 1 month, main reason for encounter					
Kroenke, 1989 [46]	USA	82	3-year incidence of the symptom of 1000 randomly selected patient files					
Valdini, 1989 [3]	USA	22	Since at least 1 year, cause unknown	X		X	X	
Kirk, 1990 [30]	USA	71	Since at least 1 month, important problem	X				
Cathebras, 1992 [2]	CDN	93	Through direct questioning (“Why did you come here today?”) or if the doctor indicated that tiredness was a reason for encounter	X		X	X	
Elnicki, 1992 [18]	USA	52	Since > 1 month, cause unknown, main reason for encounter	X		X	X	X
Gerber, 1992 [14]	USA	88	Tiredness as one of the chief complaints				X	
Ridsdale, 1993 [47]	GB	220	“being knackered, lethargic, run down or tired all the time”, since at least 2 weeks, main reason for encounter	X	X	X		
Fuhrer, 1994 [5]	F	287	Since at least 2 weeks				X	
Hall, 1994 [19]	GB	197	Diagnosis of tiredness in the patient’s file				X	
Maeno, 2002 [20]	J	157	“General fatigue”, main reason for encounter				X	
Andrea, 2003 [48]	NL	322	“Fatigue-related visite”			X		
Darbishire, 2003 [11]	GB	141	Since at least 6 months, cause unknown				X	X
Gialamas, 2003 [49]	AUS	342	“Tiredness”, “fatigue”, “weariness”, “weakness”, “lethargy” or “malaise” in patient’s record	X		X		
Kenter, 2003 [21]	NL	10.297	Episodes of care with tiredness as the reason for encounter	X			X	
Vital Durand, 2004 [50]	F	120	Since at least 6 months, cause unknown					
Belanger, 2005 [51]	CDN	36	“Fatigue” (no precise definition)					
Kenter, 2007 [12]	NL	385	Diagnosis “tiredness”				X	
Koch, 2009 [35]	NL	296	New symptom, cause unknown	X		X		
Nijrolder, 2009 [22]	NL	571	New symptom (no encounter due to tiredness in the last 6 months), main reason for encounter	X	X	X	X	X

### Quality of included studies

Nineteen of the included studies had a prospective design, 5 were retrospective analyses of clinical records and 2 were based on registries. In 15 studies consecutive recruitment was mentioned explicitly. The inclusion and exclusion criteria were well-defined in most of the studies (*n =* 24). Only 11 studies were conducted in more than one health care facility. The number of drop-outs and the reasons for withdrawal were specified in 12 studies. In 20 studies the diagnostic categories were well-defined. In 21 studies the reference standards to detect underlying illnesses were appropriate. Participating patients were subjected to the same reference standard in 12 studies. Exactly the same diagnostic investigations were applied to the patients in 8 studies. Six of the 26 studies had a control group of non-fatigued patients. Details are shown in Table 2.

**Table 2:** Methodical quality of the included studies

+ Yes	Was the recruitment of patients prospective?	Where all patients with the symptom included in the study?	Are inclusion and exclusion criteria precisely defined?	Was the study design multicentered?	Were the number of drop-outs and the reasons for withdrawal specified?	Were the diagnostic categories well-defined?	Was the reference standard that was used appropriate?	Was every patient subjected to the reference standard?	Were the same diagnostic procedures applied to all patients?	Was there an appropriate comparison group?
− No										
? Unclear										
+/−Criteria fulfilled for part of the aetiologies										
Andrea, 2003 [48]	+	+	+	−	−	?	−	−	−	+
Belanger, 2005 [51]	+	+	+	+	?	+	+	+	+	−
Cathebras, 1992 [2]	+	+	+	−	−	?	+	+	+/−	+
Darbishire, 2003 [11]	+	?	+	+	?	+	+	+/−	+/−	−
Elnicki, 1992 [18]	+	+	+	−	−	+	+	+	+/−	−
Friedlander, 1962 [43]	+	?	−	−	−	+	−	−	−	−
Fuhrer, 1994 [5]	+	+	+	+	?	+	+	+	+	+
Gerber, 1992 [14]	+	+	+	−	+	+	+	+	+	−
Gialamas, 2003 [49]	−	−	+	+	+	+	+	+/−	+/−	−
Hall, 1994 [19]	−	−	−	−	−	+	+c	?	?	−
Jerrett, 1981 [16]	+	+	+	−	−	+	+/−	+/−	+/−	−
Kenter, 2003 [21]	−	+	+	+	+	+	+	−	−	+
Kenter, 2007 [12]	+	+	+	+	?	+	+	+	+	+
Kirk, 1990 [30]	+	+	+	+	+	?	+	+	+	−
Knottnerus, 1986 [24]	+	?	+	−	+	+	+	+	+	+
Koch, 2009 [35]	+	+	+	+	+	+	+	+	+	−
Kroenke, 1989 [46]	−	?	+	−	+	?	+	−	−	−
Maeno, 2002 [20]	+	+	+	−	+	+	+	+	+	−
Morrell, 1972 [29]	+	+	+	−	+	?	+/−	−	−	−
Morrison, 1980 [15]	−	?	+	−	+	+	+	−	−	−
Nelson, 1987 [45]	+	?	+	+	−	?	−	−	−	?
Nijrolder, 2009 [22]	+	?	+	+	+	+	+	−	−	−
Ridsdale, 1993 [47]	+	?	+	+	−	+	+	+/−	+/−	−
Sugarman, 1984 [17]	−	+	+	−	+	+	+	+/−	+/−	−
Valdini, 1989 [13]	+	?	+	−	−	+	+	+	+/−	−
Vital Durand, 2004 [50]	+	+	+	?	−	+	+	+	−	−

### Tiredness in primary care: underlying diseases

**Fig. 2:**
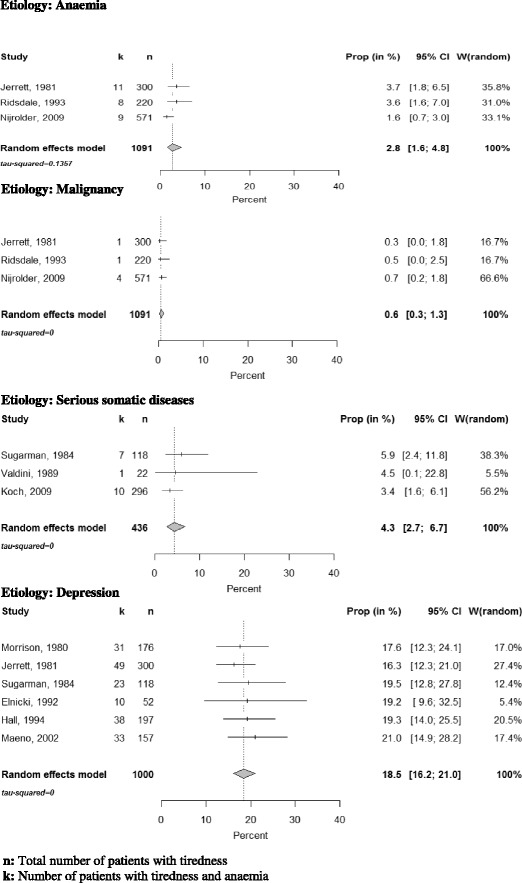
Quantitative analysis of prevalence rates of anaemia, malignancies, severe somatic diseases and depression among patients presenting with fatigue in primary care of selected studies

We present results for the following etiological categories: anaemia, malignancies, serious somatic diseases, depression and CFS (Fig. 2).

**Fig. 3:**
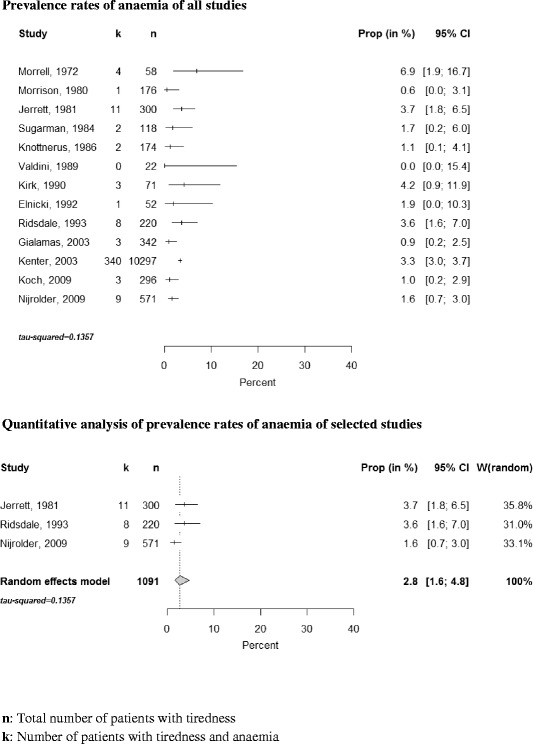
Forest plot, frequencies of anaemia and quantitative analysis

Thirteen studies assessed the frequency of anaemia in the respective study population. The prevalence rates ranged from 0 to 6.9 %; see Fig. 3. Three studies used precisely defined diagnostic criteria and an appropriate diagnostic work-up. The pooled estimate within this group was 2.8 % (95 % CI: 1.6–4.8) (see Fig. 3).

Knottnerus et al. [12] reported the prevalence of anaemia in a control group and, interestingly, found no significant difference between tired patients and those consulting for other reasons. The test used a type 1 error of level alpha of 0.05 and had a power (1-ß) of 0.09 to find a difference of 0.5 mmol/l in the mean haemoglobin level.

Three studies reported the prevalence of malignancies among patients complaining of tiredness. The prevalence rate of malignancies in our quantitative synthesis was 0.6 % (CI 0.3–1.3 %) (see Fig. 2). Jerrett, who diagnosed a carcinoma of the lung in one patient, points out that the patient’s main complaints were cough and dyspnea. Tiredness was only an additional complaint.

**Fig. 4:**
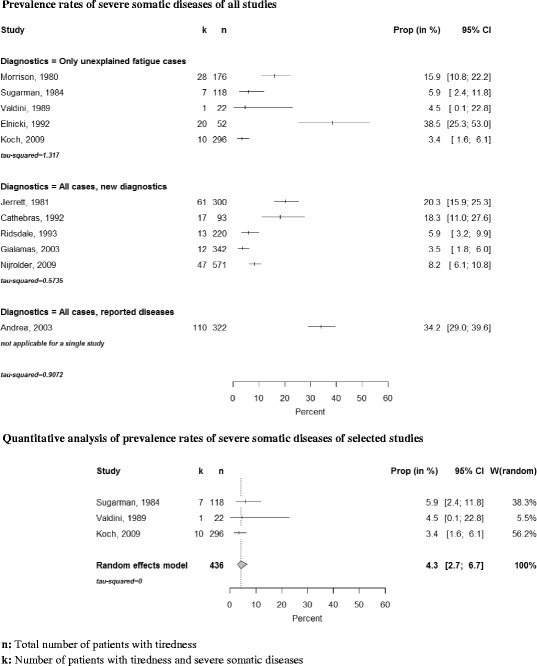
Forest plots, frequencies of severe somatic diseases and quantitative analysis

Eleven studies analysed the frequency of serious somatic diseases (see Fig. 4). In this category we included anaemia, malignancies and other serious illnesses associated with tiredness for which treatments are available. We conducted a meta-analysis with 3 studies that had prospective study designs and used defined diagnostic standards. The prevalence rate of serious somatic diseases was 4.3 % (CI 2.7–6.7 %) (see Fig. 3).

Diabetes, anaemia and hypothyroidism were most frequent. In the study of Cathebras et al. [2] serious somatic illnesses did not occur more frequently among the fatigued than among the non-fatigued study population.

**Fig. 5:**
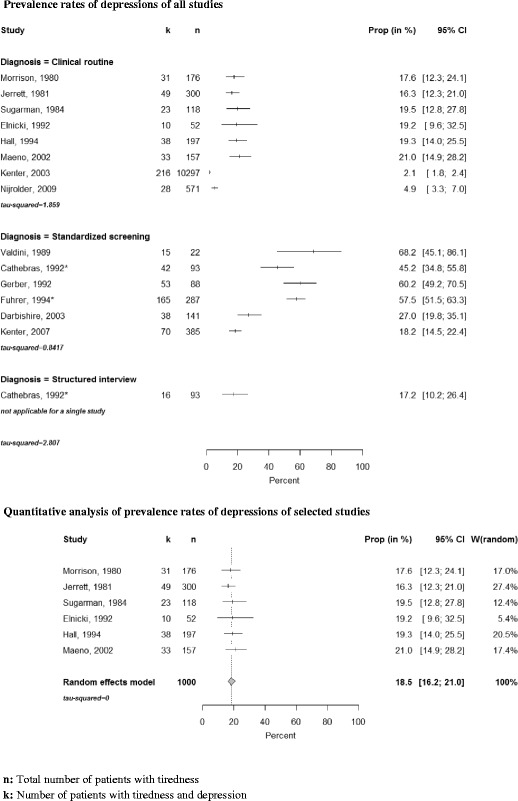
Forest plots, frequencies of depressions and quantitative analysis

Depression was investigated in 14 studies (see Fig. 5). Six studies used standardized instruments (HADS (Hospital Anxiety and Depression Scale) [11, 12], SCL-90 (Symptom Check List) [13, 14], CES-D (Center for Epidemiological Studies Depression Scale) [2, 5]). These latter studies found that up to 68.2 % of the fatigued study population scored positive. However, because of somatic and mental comorbidities these estimates may be too high. In the study of Cathebras et al. [2] all recruited patients underwent a structured interview (DIS (Diagnostic Interview Schedule). Here about 17 % (CI 10.2–26.4 %) of the patients presenting with tiredness were classified as being depressive. Most studies relied on GPs diagnosing depression according to their routine [15–22].

We performed a quantitative synthesis of 6 of these studies. Current depression was identified in 18.5 % (CI 16.2–21.0 %) of the patients (see Fig. 2). Three studies, which were not included in the meta-analysis for methodological reasons, found a significantly higher prevalence of depression in fatigued compared to non-fatigued patients [2, 5, 12].

Three studies presented estimates of the frequency of CFS among fatigued primary care attenders. Two [18, 22] reported low rates of 1.9 % (CI 0.0–10.3 %) and 0.7 % (CI 0.2–1.8 %) (see Fig. 2). Darbishire et al. [11] who used the CDC (Centers for Disease Control and Prevention) definition [23] reported a high rate of 31.2 % (CI 23.7–39.5 %). They only included patients who had suffered from tiredness for at least 6 months and had not received a diagnosis associated with tiredness. Hence two important diagnostic criteria of CFS were met by all included patients, which explains the high prevalence. Given the small number of studies we did not pool results.

## Discussion

### Summary

We identified 26 studies which investigated the underlying causes of tiredness in primary care. Among these, the most common differential diagnosis was depression (18.5 % (CI 16.2–21.0 %)). Serious somatic diseases occurred rarely (3.1 % (CI 2.7–6.7 %)). Anaemia and malignancies were present with (2.8 % (CI 1.6–4.8 %)) and (0.6 % (CI 0.3–1.3 %)) respectively. Less than 2 % of the patients were diagnosed with CFS.

### Strengths and limitations

The internal validity of some studies was limited, due to e.g. incomplete recruitment and lack of a control group. We confronted this problem by defining 10 quality criteria which were based on a proposal by Donner-Banzhoff et al. [7].

Studies differed regarding clinical characteristics, such as definition of tiredness, composition of the study population (e.g. gender, age, socio-economic status), country and health care system. The methodologies (e.g. recruitment method, diagnostic work-up, follow-up) also varied. We accounted for these differences by analysing the heterogeneity and forming subgroups. In view of the remaining heterogeneity we applied the random effects model as it accounts for variance between studies that is not due to random error.

Although our analysis provides an estimate of how frequently primary care practitioners may find anaemia in their patients, there remains the question of causality. Doubts regarding causality are fed by a comparative study with patients not having the symptom [24]. Population- and tertiary care based studies have also not found evidence for higher frequencies of anaemia among fatigued patients [25–28]. Findings from studies with control groups shed some light on the problem of causality in the clinical setting. If the prevalence of the disease under consideration does not differ between tired and non-tired patients, doubts may arise whether there is a causal association between symptom and diseases found by clinicians. Due to selective ordering of tests clinicians may be convinced that anaemia is causing tiredness in a considerable number of cases. Control group evidence, however, suggests otherwise. Similar considerations may apply also to other often non-specific symptoms.

Two outlier studies reported comparably high prevalences of anaemia (6.9 %, 4.2 %). Morrell’s study [29] had a small sample size (*n =* 58), resulting in a wide CI (1.9–16.7 %); there was no systematic reference procedure, control group nor follow-up. In Kirk et al. [30] more than one cause could be attributed to tiredness, which may have led to an overestimation.

Studies show that the majority (about 70 %) of patients suffering from a malignancy complain about tiredness [31]. In this group, tiredness often represents the most severe grievance [32]. In our systematic review, however, we examined the reverse question and found that only a very small proportion of the fatigued patients suffered from malignancies. A representative study [33], too recent to be included in our review, found a low positive predictive value (1.8 %) for the likelihood of a malignancy for fatigued patients in primary care. For tiredness as an isolated symptom the probability of having or developing a malignancy within the following year was less than 1 %. Maybe even this small estimate is too high due to a possible publication bias.

Tiredness is often an accompanying grievance of serious somatic diseases. In a British survey 50 % of chronically ill patients suffered from tiredness [34]. The majority of the included studies suggest that extensive diagnostic testing in all fatigued patients in primary care is not warranted. Excessive testing will lead to a considerable number of false-positive tests and rarely help detect serious diseases if fatigue occurred as an isolated symptom without additional abnormalities in the medical history and in the clinical examination [13, 35].

Numerous studies indicate the strong association between psychiatric disorders and tiredness. Approximately 75 % of people suffering from a depression indicate tiredness as a central grievance [36]. Our systematic review proves the close relationship between tiredness and depression among primary care attenders. The co-occurrence varies depending on the definitions of tiredness and depression. Given the high level of comorbidity in primary care samples, we restricted our analysis to studies which reported family physicians’ diagnosis of depression. A similar proportion of depressed patients 17.2 % (CI 10.2–26.4 %) was reported by a study employing a structured interview schedule [2].

There is an ongoing controversy about the question whether CFS can be considered as a distinct clinical entity [37, 38]. There are different case definitions and the pathophysiology and the aetiology of CFS remain unclear. The prevalence of CFS found in community-based studies is low; a meta-analysis of 5 studies found a frequency of 0.87 % [39]. A British study [40] with a large primary care sample of 143,000 patients found a prevalence rate of 0.2 %. Even a study which has been carried out in a clinic specializing in fatigue found a relatively low prevalence rate of 4 % [41].

## Conclusions

About every fifth patient complaining of tiredness to the GP (general practitioner) suffers from a depressive disorder. A history targeted at mental health and psycho-social well-being is therefore of great importance. Our review shows that anaemia, malignancies and other serious somatic diseases are only very rarely found in fatigued primary care patients. Their prevalence rates hardly differ from non-fatigued patients. Extensive investigations are not indicated if tiredness occurs as an isolated symptom without additional findings from the history or physical examination. Results of this meta-analysis have been included in the Practice Guideline of the German College of General Practitioners and Family Physicians [42].

## Additional files

Additional file 1: Search syntax for Pubmed. (DOCX 16 kb)
Additional file 2: Detailed characteristics of the included studies. (DOCX 31 kb)

## Abbreviations

CDC: Centers for disease control and prevention
CES-D: Center for epidemiological studies depression scale
CFS: Chronic fatigue syndrome
CI: Confidence interval
DIS: Diagnostic interview schedule
GP: General practitioner
HADS: Hospital anxiety and depression scale
SCL-90: Symptom check list
