# Supplementary material for: The differential diagnosis of tiredness: a systematic review
Source: BMC Fam Pract. 2016 Oct 20;17:147. doi: 10.1186/s12875-016-0545-5 (PMC5072300; doi:10.1186/s12875-016-0545-5)
Supplement: Additional file 1: — Search syntax for Pubmed. (DOCX 16 kb) [file 12875_2016_545_MOESM1_ESM.docx]

# Additional file 1: Search syntax for Pubmed

| **Symptom in various notations**  **(in title and/ or abstract)** | Fatigue [TIAB]  Tired [TIAB]  Tiredness [TIAB]  Exhaustion [TIAB]  Exhausted [TIAB]  Weary [TIAB]  Weariness [TIAB]  Lethargy [TIAB]  Lethargic [TIAB] | |
| --- | --- | --- |
| OR | | |
| **Symptom as the MESH term** | Fatigue [Mesh]  “Fatigue Syndrome, chronic” [Mesh]  “Muscle Fatigue” [Mesh] | |
| AND | | |
| **Term „general practice“ in various notations (in title and/ or abstract)** | "general practitioner” [TIAB]  “general practitioners” [TIAB]  “general practice” [TIAB]  “family practice” [TIAB]  “family practitioners” [TIAB]  “family practitioner” [TIAB]  “family medicine” [TIAB]  “family physician” [TIAB]  “family physicians” [TIAB]  “family doctor” [TIAB]  “family doctors” [TIAB]  “primary care” [TIAB] | OR |
| OR | | |
| **Journal representing our research field** | "BMC Fam Pract" [TA]  "Fam Pract" [TA]  "J Fam Pract" [TA]  "Fam Pract Res J" [TA]  "J Am Board Fam Pract" [TA]  "Br j gen pract" [TA]  “J R Coll Gen Pract” [TA]  “J Coll Gen Pract” [TA]  “J Coll Gen Pract Res Newsl” [TA]  "Can fam physician" [TA]  "Ann Fam Med" [TA]  "Aust fam physician" [TA]  "Scand J Prim Health Care" [TA]  "Eur J Gen Pract" [TA]  "Archives of family medicine" [Journal]  “J Gen Intern Med” [TA] "Atencion primaria / Sociedad Española de Medicina de Familia y Comunitaria" [Journal] | OR |
| OR | | |
| **Term „general practice“ in various notations (in affiliation to authors)** | “general practice” [AD]  “family practice*” [AD]  “family medicine” [AD]  “primary care” [AD]  community [AD] | OR |
| OR | | |
| **MESH terms “family practice”,**  **“physicians, family” and “primary health care“** | "Family Practice" [Mesh]  "Physicians, Family" [Mesh]  "Primary Health Care" [Mesh] | OR |

Limits: NOT: Editorial, Meta-Analysis, Practice Guideline, Review, Addresses, Bibliography, Biography, Case Reports, Comment, Dictionary, Directory, Festschrift, Government Publications, Guideline, Historical Article, In Vitro, Interactive Tutorial, Interview, Legal Cases, Legislation, Patient Education Handout, Portraits, Webcasts
