# Supplementary material for: The differential diagnosis of tiredness: a systematic review
Source: BMC Fam Pract. 2016 Oct 20;17:147. doi: 10.1186/s12875-016-0545-5 (PMC5072300; doi:10.1186/s12875-016-0545-5)
Supplement: Additional file 2: — Detailed characteristics of the included studies. (DOCX 31 kb) [file 12875_2016_545_MOESM2_ESM.docx]

| **First author, year** | **Country** | **Sample size** | **1. Primary symptom or among symptoms (=one of the mentioned symptoms, not necessarily the primary symptom)**  **2. Symptomdefinition** | **1.: Inclusion criteria**  **2.: Exclusion criteria** | **Data assessment** | **Anaemia** | **Malignancies** | **Severe somatic diseases** | **Depressions** | **Chronic fatigue syndrom** | **Somatic causes** | **Psychological causes** | **Anxiety disorder** |
| --- | --- | --- | --- | --- | --- | --- | --- | --- | --- | --- | --- | --- | --- |
| Friedlander, 1962 | USA | 71 | 1. Primary symptom  2. "Unbearable fatigue" | 1. „Unbearable fatigue“ | Prospective |  |  |  |  |  | X | X |  |
| Morrell, 1972 | GB | 58 | 1. Among symptoms  2. New symptom that wasn’t brought up at a doctor’s visit during the last 12 months | 1. All patient- initiated visits  2. From the doctor initiated visits | Prospective | X |  |  |  |  |  | X |  |
| Morrison, 1980 | USA | 176 | 1. Among symptoms  2. Cause unknown | 1. Cause unknown  2. Diagnosis of mononucleosis | Retrospective | X |  | X | X |  |  |  |  |
| Jerrett, 1981 | GB | 300 | 1. Among symptoms  2. Tiredness as a central problem ("tiredness", "need of tonic", "run down", etc.) | 1. Tiredness as a main or subsidiary reason for encounter | Prospective | X | X | X | X |  |  |  |  |
| Sugarman, 1984 | USA | 118 | 1. Among symptoms  2. Cause unknown | 1. > 14 y., diagnosis: „fatigue, tiredness or malaise“  2. Known cause of tiredness at initial encounter | Retrospective | X |  | X | X |  |  |  |  |
| Knotterus, 1986 | NL | 174 | 1. Among symptoms  2. New Symptom | 1. > 17 y.  2. Pregnancy, iron substitution, cause appart from anaemia probable at first encounter | Prospective | X |  |  |  |  |  |  |  |
| Nelson, 1987 | USA | 71 | 1. Primary symptom  2. At least 1 month | 1. >17 Y., tiredness as a  main reason for encounter | Prospective |  |  |  |  |  | X | X |  |
| Kroenke, 1989 | USA | 82 | 1. Among symptoms  2. 3-year incidence of the symptom of 1000 randomly selected patient files | 1. Patients visiting the practice from 1984-1987 | Retrospective |  |  |  |  |  | X | X |  |
| Valdini, 1989 | USA | 22 | 1- Among the symptoms  2. Since at least 1 year, cause unknown | 1. Tiredness since > 1 y., cause unknown | Prospective | X |  | X | X |  |  |  |  |
| Kirk, 1990 | USA | 71 | 1. Among symptoms  2. Since at least 1 month, important problem | 1. > 17 y., tiredness since at least 1 month and as an central problem | Prospective | X |  |  |  |  | X | X |  |
| Cathebras, 1992 | CN | 93 | 1. Among symptoms  2. Direct questioning („Why did you come here today?“) or if the doctor indicated that tiredness was a reason for encounter | 1. 18-75 y., new medical problem, sufficient command of English or French | Prospective | X |  | X | X |  |  |  | X |
| Elnicki, 1992 | USA | 52 | 1. Primary symptom  2. Tiredness main p­­­­­roblem, since > 1 month, cause unknown | 1. Tiredness as a main problem since > 1 month  2. Known psychological illness, diagnosis of severe somatic illness | Prospective | X |  | X | X | X | X |  |  |
| Gerber, 1992 | USA | 88 | 1. Primary symptom  2. No specific definition | 1. > 17 y. | Prospective |  |  |  | X |  |  |  |  |
| Ridsdale, 1993 | GB | 220 | 1. Primary symptom  2. "being knackered, lethargic, run down or tired all the time", since at least 2 weeks | 1. > 15 y., tiredness as the main reason for encounter, since at least 2 weeks | Prospective | X | X | X |  |  |  |  |  |
| Fuhrer, 1994 | F | 287 | 1. Among symptoms  2. Since at least 2 weeks | 1. 18-64 y. | Prospective |  |  |  | X |  |  |  |  |
| Hall, 1994 | GB | 197 | 1. Among symptoms  2. Diagnosis or tiredness in the patient‘s file | Specific inclusion or exclusion criteria are not mentioned | Retrospective |  |  |  | X |  | X |  | X |
| Maeno, 2002 | J | 157 | 1. Among symptoms | 2. Pat. with an obvious medical condition | Prospective |  |  |  | X |  |  |  |  |
| Andrea, 2003 | NL | 322 | 1. Among symptoms  2. "Fatigue-related visite" | 1. Employees from one of the recruiting companies  2. Employees not actively at work, missing values concerning work status | Prospective |  |  | X |  |  | X |  |  |
| Darbishire, 2003 | GB | 141 | 1. Among symptoms  2. Since at least 6 months, cause unknown | 1. 16-75 y., fatigue as a major problem, since at least 6 months, no change of drug regime, normal blood exams  2. Psychotic illness, organic brain syndrome, substance dependency, current psychiatric treatment, concurrent physical problem that could have caused fatigue | Prospective |  |  |  | X | X | X | X | X |
| Gialamas, 2003 | AUS | 342 | 1. Among symptoms  2. "Tiredness", "fatigue", "weariness", "weakness", "lethargy" or "malaise" in patient’s record | 2. < 5 y. of fatigue | Retrospective | X |  | X |  |  |  |  |  |
| Kenter, 2003 | NL | 10.297 | 1. Primary symptom  2. Episodes of care starting with the reason for encounter "tiredness" | 1. Patients listed for the full 16-year period | Register data | X |  |  | X |  |  |  |  |
| Vital Durand, 2004 | F | 120 | 1. Among symptoms  2. Since at least 6 months, cause unknown | 1. > 18 y., tiredness since at least 6 months, cause unknown  2. Somatic or mental disorder that could cause tiredness, depression | Prospective |  |  |  |  |  |  |  |  |
| Belanger, 2005 | CDN | 36 | 1. Primary symptom | 1. > 18 y., being able to fill out questionnaire | Prospective |  |  |  |  |  |  |  | X |
| Kenter, 2007 | NL | 385 | 1. Among the symptoms | 1. > 15 y., tiredness as the reason for encounter, diagnosis “tiredness” given | Register data |  |  |  | X |  |  |  | X |
| Koch, 2009 | NL | 296 | 1. Among symptoms  2. New symptom, cause unknown | 1. > 17 y., new symptom, sufficient command of Dutch | Prospective | X |  | X |  |  |  |  |  |
| Nijrolder, 2009 | NL | 571 | 1. Primary symptom  2. New symptom (no encounter due to tiredness in the last 6 months) | 1. New and main symptom  2. Chemotherapie, radiotherapy, pregnancy, post-partum | Prospective | X | X | X | X | X | X | X | X |
